# Kamishoyosan Normalizes Dendritic Spine Morphology in the Medial Prefrontal Cortex by Regulating microRNA-18 and Glucocorticoid Receptor Expressions in Postmenopausal Chronic Stress-Exposed Mice

**DOI:** 10.7759/cureus.63526

**Published:** 2024-06-30

**Authors:** Shoko Shimizu, Yoshihisa Koyama, Yugo Ishino, Takashi Takeda, Shoichi Shimada, Masaya Tohyama, Shingo Miyata

**Affiliations:** 1 Molecular Brain Science, Research Institute of Traditional Asian Medicine, Kindai University, Osaka-Sayama, JPN; 2 Neuroscience and Cell Biology, Osaka University Graduate School of Medicine, Suita, JPN; 3 Women Medicine, Research Institute of Traditional Asian Medicine, Kindai University, Osaka-Sayama, JPN; 4 Operations, Osaka Prefectural Hospital Organization, Osaka, JPN

**Keywords:** medial prefrontal cortex, spine morphology, glucocorticoid receptor, microrna, hypothalamic-pituitary-adrenal axis, menopause, kamishoyosan

## Abstract

Objective: Kamishoyosan (KSS), a traditional Japanese Kampo medicine, is widely used to treat neuropsychiatric symptoms in perimenopausal and postmenopausal women. We aimed to elucidate the functional mechanisms underlying KSS-mediated reduction of stress response behaviors and neuropsychological symptoms in perimenopausal and postmenopausal women.

Methods: Female mice were bilaterally ovariectomized (OVX) at the age of 12 weeks and exposed to chronic water immersion and restraint stress for three weeks. Among them, mice in the OVX+stress+KSS group were fed chow containing KSS from one week before exposure to chronic stress until the end of the experiment. Firstly, we performed a marble burying test and measured serum corticosterone levels to assess irritability and stress conditions. Next, we examined whether KSS affects microRNA-18 (miR-18) and glucocorticoid receptor (GR) protein expression, as well as the basal dendritic spine morphology of pyramidal neurons in the medial prefrontal cortex (mPFC) of postmenopausal chronic stress-exposed mice. Analyzed data were expressed as mean ± standard deviation. Tukey’s post hoc test, followed by analysis of variance (ANOVA), was used for among-group comparisons.

Results: KSS administration normalized chronic stress-induced unstable emotion-like behavior and upregulated plasma corticosterone levels. Furthermore, KSS ameliorated GR protein expression by downregulating miR-18 expression in the mPFC and recovered the immature morphological changes in spine formation of pyramidal neurons in the mPFC of OVX mice following chronic stress exposure.

Conclusions: KSS administration in postmenopausal chronic stress-exposed mice exerted anti-stress effects and improved the basal dendritic spine morphology of pyramidal neurons by regulating miR-18 and glucocorticoid receptor expression in the mPFC.

## Introduction

Menopause-related neuropsychological symptoms, including irritation, depression, and anxiety, are characterized by cognitive, autonomic, emotional, and endocrine function disturbances [1]. One of the traditional Japanese Kampo medicines, Kamishoyosan (KSS), is composed of 10 crude compounds containing a specified mixture derived from plant sources and is widely prescribed to improve various neuropsychiatric symptoms in perimenopausal and postmenopausal women [2,3]. A previous KSS clinical study in postmenopausal women and a premenstrual rat model reported that KSS administration caused significant improvements in excitability and irritability scores [4,5]. Apart from these studies, we previously demonstrated that continuous KSS administration in postmenopausal chronic stress-exposed mice attenuated stress-related depressive behavior and normalized hypothalamic-pituitary-adrenal (HPA) axis activity [6]. However, the molecular mechanisms underlying the beneficial effects of KSS-mediated regulation of the HPA axis remain unclear.

The prefrontal cortex (PFC), particularly the medial prefrontal cortex (mPFC) in humans, is critical to higher-order executive functions, memory, decision-making, cognition, and emotional control [7]. The mPFC is vulnerable to stress, which can decrease its volume and synaptic density by changing spine morphology in patients with depression [8]. Decreased PFC activity is closely related to the onset of stress-related psychiatric diseases such as depression [9]. Therefore, prefrontal hypofunction induced by stress exposure is strongly implicated in the onset of psychiatric symptoms. However, the molecular mechanisms underlying menopause-related neuropsychological symptoms in mPFC functions remain unclear.

Glucocorticoid receptors (GRs) are also distributed in the PFC, and prefrontal GRs have been recently implicated in HPA axis regulation and mood regulation [10,11]. These studies suggest a functional association between defective prefrontal GR signaling and stress-related psychiatric diseases. Furthermore, a previous study indicated that elevated microRNA-18 (miR-18) expression and reduced GR protein expression were reported in the paraventricular hypothalamic nucleus of stress-vulnerability model rats [12]. However, the functions of miR-18 in the mPFC of postmenopausal environmental stress-exposed mice remain unclear.

Accordingly, as a continuation of our previous KSS research, we aimed to evaluate the effects of KSS on miRNA-mediated regulation of GR expression in the mPFC and the basal dendritic spine morphology of pyramidal neurons in the mPFC of postmenopausal environmental stress-exposed mice in this study.

## Materials and methods

Ethics statement

All animal experiments were conducted according to the Guiding Principles for the Care and Use of Laboratory Animals, the United States National Institutes of Health Guide for the Care and Use of Laboratory Animals, and the animal care and handling procedures approved by the International Animal Care and Use Committee of Kindai University (No. KAME-25-009).

Animals

Ten-week-old C57BL/6N female mice were purchased from SLC (Japan SLC, Inc., Hamamatsu, Japan). Three mice per cage were kept at room temperature (22 ± 2°C; humidity, 55 ± 10%) in a 12-h light/dark cycle (lights on at 07:00 a.m. and off at 07:00 p.m.). The animals had free access to water and food for breeding (CE-2; CLEA Japan Inc., Tokyo, Japan).

KSS administration and stress exposure

KSS is composed of the extractions of 10 medicinal herbs [6] (Table 1) and was supplied by Tsumura & Co. (Tokyo, Japan). These ingredient content percentages were calculated from the KSS product label (Tsumura & Co.). Dry powdered extracts of KSS were mixed with CE-2 chow at a final concentration of 3% (w/w) and used as previously reported [6].

**Table 1:** Composition of Kamishoyosan

Ingredient	Content (%)
Bupleuri Radix (*Bupleurum falcatum*)	13.3
Paeoniae Radix (*Paeonia lactiflora*)	13.3
Atractylodis Rhizoma (*Atractylodes ovate*)	13.3
Angelicae Radix (*Angelica acutiloba*)	13.3
Hoelen (*Poria cocos*)	13.3
Gardeniae Fructus (*Gardenia jasminoides*)	8.9
Moutan Cortex (*Paeonia suffruticosa*)	8.9
Glycyrrhizae Radix (*Glycyrrhizae uralensis*)	6.7
Zingiberis Rhizoma (*Zingiber officinale*)	4.4
Menthae Herba (*Menthae arvensis*)	4.4

All female mice were bilaterally ovariectomized (OVX) at age 12 weeks. After two weeks of postoperative recovery, the 36 mice were randomly allocated into three groups (n=12) after the ovariectomy: the control group (non-stressed OVX mice), the chronically stressed group (OVX+Stress mice), and the chronically stressed group administered with KSS (OVX+Stress+KSS mice). There was no significant difference in body weight and average daily consumption of chow between groups before stress exposure. Chronic stress was induced as previously described [6]. Briefly, OVX mice were exposed to chronic Water Immersion and Restraint Stress (WIRS) for three weeks (at 14-17 weeks of age). Specifically, they were restrained in a 50-mL conical polypropylene centrifuge tube and vertically immersed to the level of the xiphoid process in a water bath maintained at 23°C for two hours once a day for three weeks. For KSS administration, mice in the OVX+Stress+KSS group were fed CE-2 chow containing 3% KSS one week before chronic stress exposure (13 weeks old) until the end of the experiment as previously reported [6]. The mice were confirmed to be free of gastric and duodenal ulcers by visual observation for bleeding and sores on the surface of the stomach and duodenal mucosa.

Enzyme-linked immunosorbent assay (ELISA)

Serum corticosterone levels were measured using a Corticosterone Enzyme Immunoassay Kit (Arbor Assays, K014, Ann Arbor, Michigan), following the manufacturer's instructions. Briefly, one day after the chronic stress exposure period, the mice were deeply anesthetized, and their blood samples were collected into tubes containing heparin. The tubes were immediately placed on ice, followed by centrifugation at 1000 g for 15 minutes at 4°C. Plasma samples were stored at -80°C prior to assays. Absorbance at 450 nm was measured using a plate reader (Multiskan FC, Thermo Fisher Scientific Inc., Waltham, Massachusetts), and the corticosterone concentration in each sample was calculated using the SkanIt™ microplate reader software (Thermo Fisher Scientific Inc.).

Marble burying test

To assess behaviors representing unstable emotions, anxiety, and irritability, a marble burying test was performed two days after the end of the chronic stress exposure period [13]. Briefly, 20 glass marbles were evenly distributed on 5-cm-deep sawdust bedding in 4 × 5 grids in standard cages (25 × 25 × 31 cm). Each mouse was placed in a cage for 15 minutes. Subsequently, the mice were removed from the cage, and the number of marbles buried by the mice was counted at the end of the test. Marbles buried to at least 2/3 of their depth were considered buried [13]. The light intensity in both the breeding and test rooms was ~150 lux at 40 cm from the floor.

Quantitative real-time polymerase chain reaction (PCR)

Total RNA was extracted from the PFC of mice using Isogen II (NipponGene, Toyama, Japan), following the manufacturer's instructions. To analyze mRNA expression, reverse transcription of the total RNA was performed using a High-Capacity cDNA Reverse Transcription Kit (Thermo Fisher Scientific Inc.). To evaluate GR and GAPDH expression, quantitative real-time PCR (qRT-PCR) was conducted using KOD SYBR qPCR Mix (TOYOBO Co., Ltd., Osaka, Japan) with the following forward/reverse primers: GR, 5’-ACCTGGATGACCAAATGACCC-3’/5’-GCATAGCAGGTTTCCACTTGC-3’ and GAPDH, 5’-GTGTTCCTACCCCCAATGTG-3’/5’-AGGAGACAACCTGGTCCTCA-3’. The housekeeping gene GAPDH was used as the internal control [6]. Specific ratio comparisons (gene of interest/GAPDH) were used to assess between-group differences in transcript expression. To analyze miRNA expression, reverse transcription of total RNA was performed using the TaqMan MicroRNA Reverse Transcription kit (Thermo Fisher Scientific Inc.) according to the manufacturer's instructions. To detect mature miR-18, qRT-PCR was performed using TaqMan Universal PCR Master Mix (Thermo Fisher Scientific Inc.). TaqMan assays specific for miR-18 (Thermo Fisher Scientific Inc.) were performed with an ABI7900HT PCR System according to the manufacturer's instructions (Thermo Fisher Scientific Inc.). The relative levels of miR-18 in the mPFC were calculated with the 2−ΔΔCT method, with U6 as an internal control.

Golgi staining

Golgi staining was performed using an FD Rapid Golgi Stain Kit (FD NeuroTechnologies Inc., Columbia, Maryland), as previously described [14]. Briefly, the brains were removed from anesthetized mice and immersed in an equal mixture of solutions A and B for three weeks at 22±2°C in the dark. Next, the brains were transferred into solution C for seven days at 22±2°C. After freezing on dry ice, 200-µm serial coronal sections of the brain samples were prepared using a cryostat at -24°C and mounted on a 0.5% gelatin-coated glass slide, incubated overnight at 22±2°C, and soaked in solution C for five minutes. Subsequently, the slides were stained with a mixture of solution D, solution E, and deionized water (1:1:2) for 10 minutes, then rinsed in distilled water twice for four minutes. Coronal sections were dehydrated in an ascending ethanol series, cleared with xylene, and sealed with Entellan (Merck KGaA, Darmstadt, Germany). All images of the pyramidal neurons in the mPFC were obtained using a Keyence microscope (Keyence Corp., Osaka, Japan).

Immunohistochemistry

Immunohistochemical staining of the mouse brain was performed as previously described [6]. Briefly, mice were perfused transcranially with 4% paraformaldehyde three days after the three-week exposure to chronic stress. Next, their brains were collected and immersion-fixed in 4% paraformaldehyde at 4°C overnight. After post-fixing, the brain tissues were stored in a 30% (w/v) sucrose solution in 0.1 M phosphate-buffered saline (PBS) for 48 hours at 4°C. Free-floating, 30-µm-thick sections were rinsed with PBS and incubated in blocking buffer (5% bovine serum albumin and 0.3% Triton X-100 in PBS) for one hour at room temperature. Subsequently, the sections were incubated with primary antibodies overnight at 4°C (Table 2). Next, the sections were washed in PBS and incubated with Alexa 488 anti-rabbit IgG secondary antibody (1:1000, Thermo Fisher Scientific Inc.; A-11008, RRID: AB_143165) for two hours at room temperature. All images were acquired using a laser scanning confocal microscope (C2; Nikon Corp., Tokyo, Japan). Immunohistochemical staining intensities were determined using ImageJ (National Institutes of Health). To quantify the GR expression level, the images were analyzed with pixel values of fluorescence intensity using the ImageJ software relative to a predetermined threshold intensity (the background intensity of the images set to zero). The same threshold setting was applied to all the images in each comparison group.

Immune blotting analyses

Immune blotting analysis was performed as previously described [6] using the antibodies in Table 2. Immunodetection of target proteins was performed using horseradish peroxidase-conjugated secondary antibodies (1:5000; Cell Signaling Technology Inc.) and an ECL Prime Western Blotting Detection System (GE Healthcare Systems Inc., Chicago, Illinois). Densitometric quantification was performed using ImageJ (National Institutes of Health), with glyceraldehyde-3-phosphate dehydrogenase (GAPDH) as the loading control. PSD95, postsynaptic density protein 95.

**Table 2:** The information about antibodies GR, glucocorticoid receptor; PSD95, postsynaptic density protein 95; GAPDH, glyceraldehyde-3-phosphate dehydrogenase; IHC, immunohistochemistry; WB; western blotting

Antibody	Code No,	Manufacturer	Dilution
GR	ab183127	Abcam Plc, Cambridge, England	1:500 (IHC)
GR	ab183127	Abcam Plc, Cambridge, England	1:1000 (WB)
PSD95	3450	Cell Signaling Technology Inc., Danvers, Massachusetts	1:1000 (WB)
GAPDH	sc-32233	Santa Cruz Biotechnology Inc., Dallas, Texas	1:1000 (WB)

Statistical analyses

Statistical analyses were performed using GraphPad Prism 10, a statistical software commonly used for data analysis in basic medical research (GraphPad Software, Boston, Massachusetts). Data are expressed as mean ± standard deviation (SD). Analysis of variance (one-way ANOVA) was used for 3 among-group statistical differences, followed by Tukey’s post hoc test. Tukey's post-hoc test is a widely used statistical method with robust power to identify significant differences between groups and handle unequal sample sizes and variances. Statistical significance was set at P < 0.05.

## Results

KSS normalized stress-upregulated plasma corticosterone level and irritability behavior

**Figure 1:**
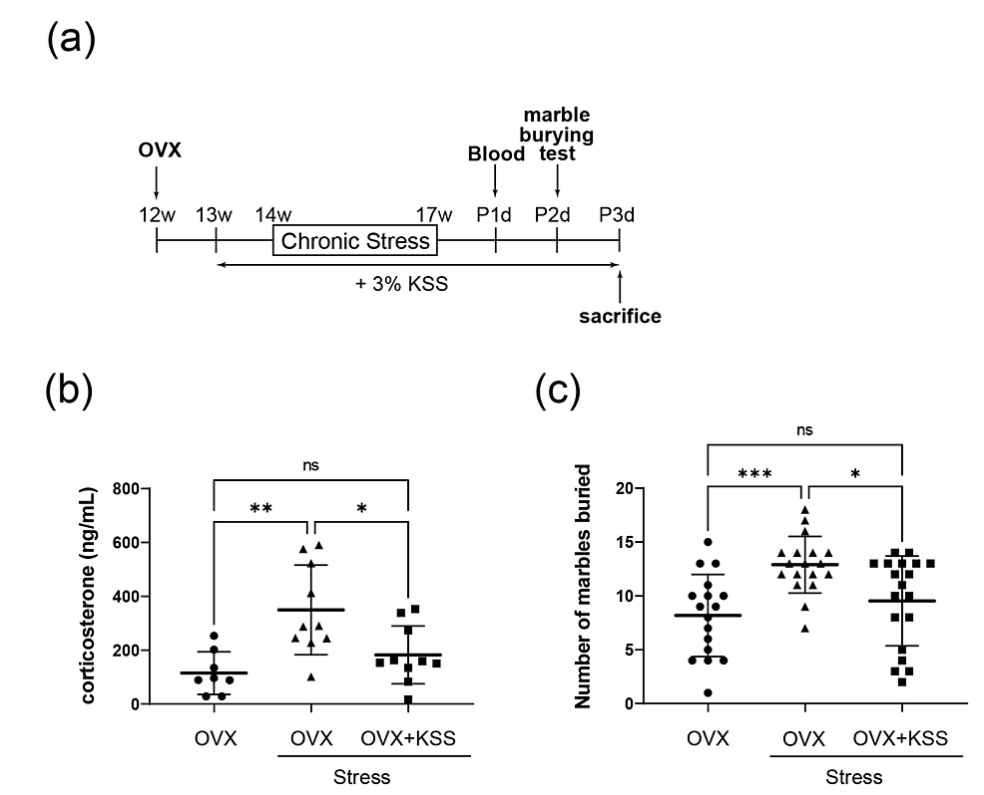
KSS treatment effects on plasma corticosterone levels and irritability-like behavior in OVX+Stress mice. (a) Experimental timeline. The mice were used from 12 w (weeks) to 17w. Several analyses were performed from P1d (post 1 day) to P3d. (b) Levels of plasma corticosterone were measured by ELISA using blood samples. Results are shown as means ±SD (n = 8-10). (c) Marble Burying Test. Chronic stress exposure increased the number of buried marbles, which was normalized by the KSS administration. Results are shown as means ± SD (n = 17-19). *P < 0.05, **P < 0.01 one-way ANOVA followed by Tukey’s post-test. KSS, Kamishoyosan; OVX, ovariectomized mice; ELISA, enzyme-linked immunosorbent assay; SD, standard deviation; ANOVA, analysis of variance; ns, no statistically significant difference.

Compared with control OVX mice, OVX+Stress mice showed upregulated plasma corticosterone levels (Figure 1a, b), whereas the levels in OVX+Stress+KSS mice resembled those of the control (Figure 1a, b). Moreover, OVX+Stress mice showed an increased number of buried marbles, which was lower in OVX+Stress+KSS mice (Figure 1c). These results suggest that KSS administration ameliorated chronic stress-induced continuous hyperactivity of the HPA axis and unstable emotional behavior in OVX+Stress+KSS mice.

Dendritic spine maturation and synaptic function in the mPFC were ameliorated from the effects of chronic stress by KSS

**Figure 2:**
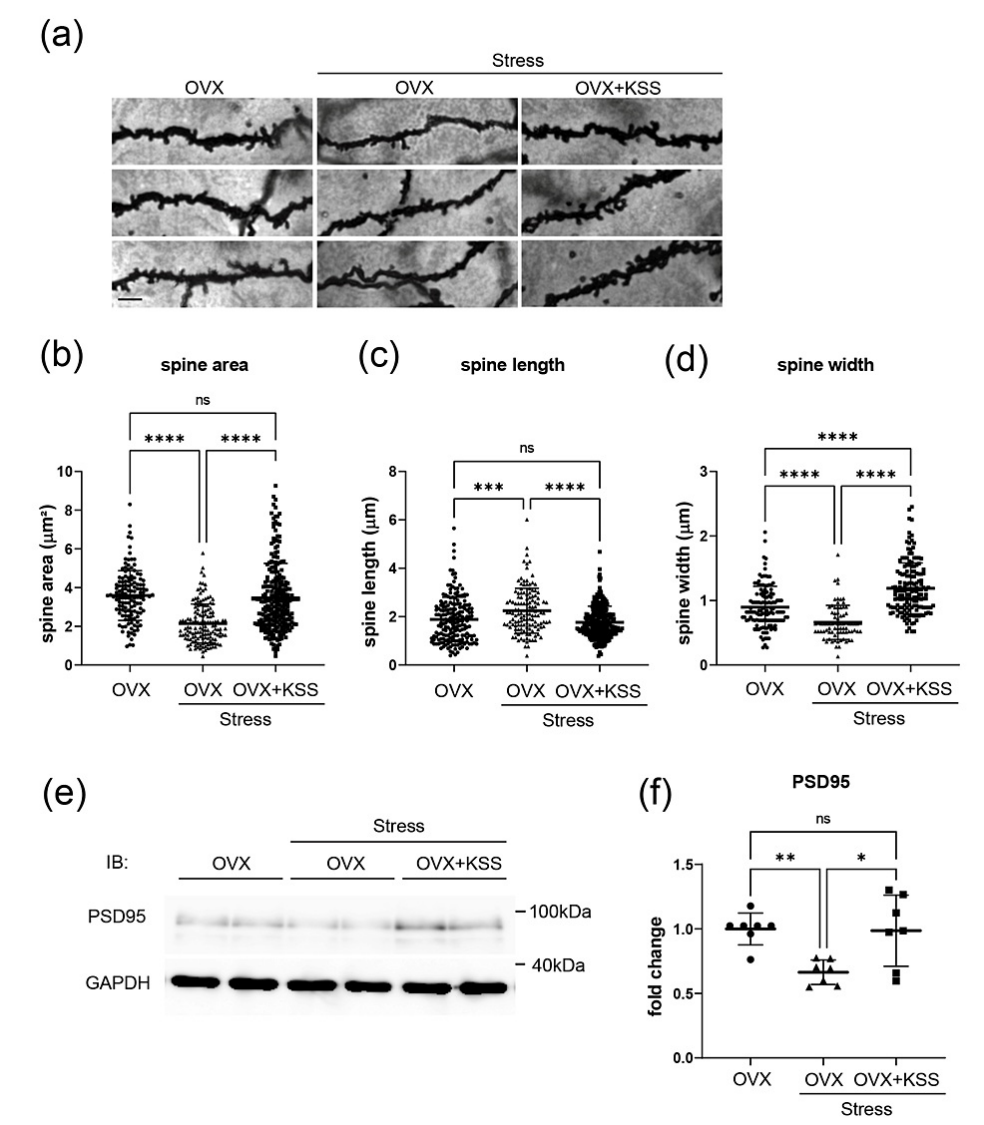
KSS treatment effects on basal dendritic spines of pyramidal neurons in the mPFC. (a) High-magnification image of the representative Golgi-stained dendritic segments of pyramidal neurons in the mPFC. Scale bar, 200 nm. (b-d) Spine area, length, and width of basal dendrites from the cell soma of pyramidal neurons in the mPFC. Data are presented as means ± SD. Two hundred spines from four slices from three animals per group were analyzed. Compared with OVX and OVX+Stress+KSS mice, OVX+Stress mice showed reduced spine area, length, and width. Data are presented as means ± SD. (e) PSD95 expression was evaluated through western blot analysis. (f) Densitometric quantification of PSD95 expression. The results are shown as means ± SD (n = 6). One-way ANOVA, Tukey’s multiple comparisons test, *p < 0.05, **P < 0.01, ***P < 0.001, ****P＜0.0001. Scale bar: 200 nm. KSS, Kamishoyosan; OVX, ovariectomized mice; mPFC, medial prefrontal cortex; IB, immune blotting; PSD95, postsynaptic density protein 95; GAPDH, glyceraldehyde-3-phosphate dehydrogenase; kDa, kilo Dalton; ns, no statistically significant difference.

Chronic stress significantly decreased the spine area and spine head width in the mPFC of OVX mice, while these changes were absent in chronic stress-exposed OVX mice administered KSS (Figures 2a, b, d). Furthermore, OVX+Stress mice showed increased spine length compared with stress-exposed OVX mice that received KSS (Figures 2a, c). To evaluate synaptic function in the mPFC, we assessed the expression level of the postsynaptic density protein 95 (PSD95). Western blot analysis revealed decreased PSD95 expression in the mPFC of OVX+Stress mice, whereas expression in OVX+Stress+KSS mice was equivalent to that of the control (Figures 2e, f). These results suggest that KSS administration ameliorated defects induced by chronic stress in spine maturation and synaptic function in the mPFC of OVX+Stress mice.

KSS recovered GR protein expression changes through miR-18 upregulation in the mPFC

**Figure 3:**
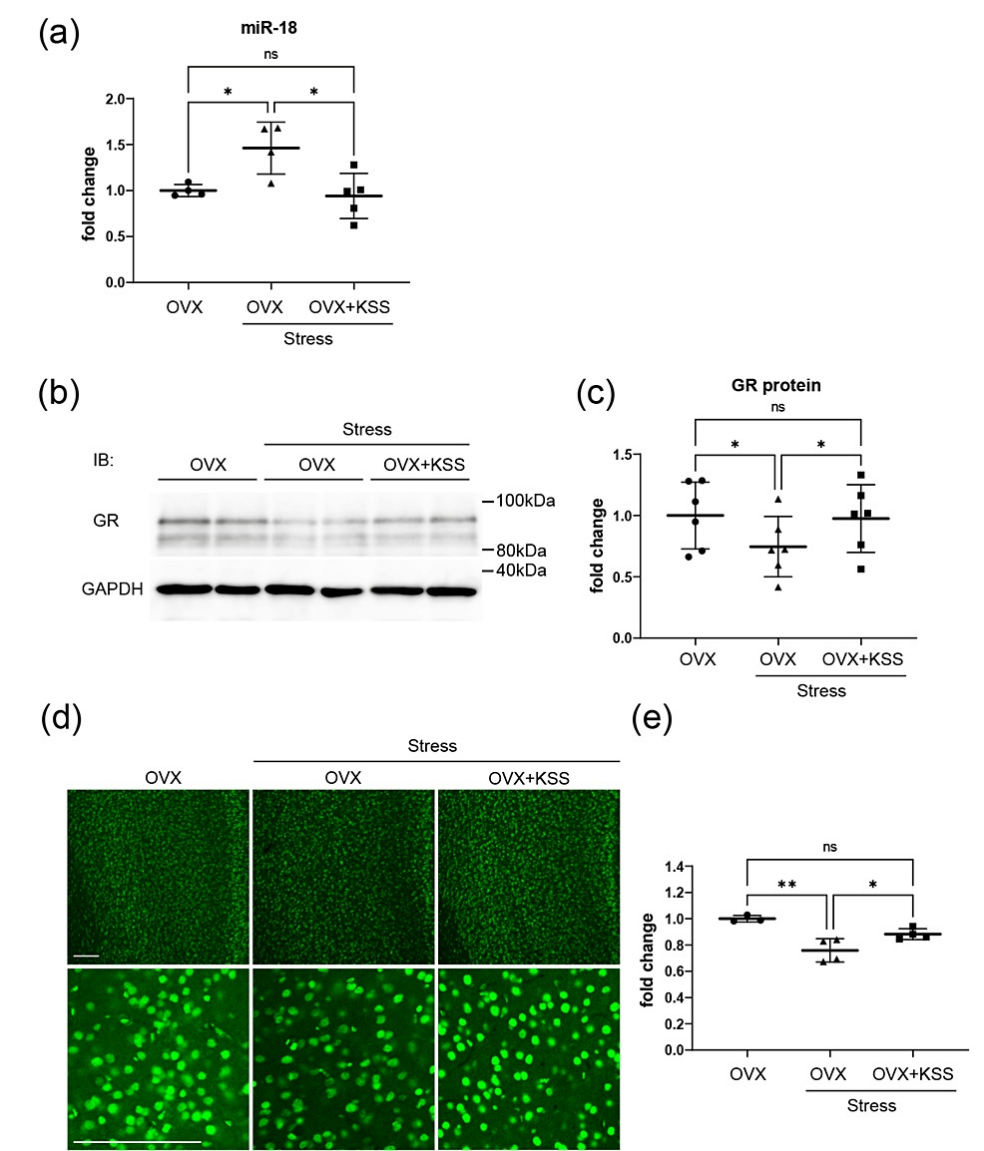
KSS treatment effects on miR-18 level and GR protein expression in the mPFC in OVX+Stress mice. (a) Expression of miR-18 in the PFC was quantified through quantitative RT-PCR. (b) GR expression was assessed through western blot analysis. (c) Densitometric quantification of GR expression. These results are shown as means ± SD (n = 6). (d) Representative staining (upper panels) and high magnification (lower panels) images of GRs (green) in the mPFC. (e) The relative fluorescence intensity of GR signals in the mPFC. These results are shown as means ± SD (n = 4). Scale bar: 100 µm. PFC, prefrontal cortex; RT-PCR, reverse transcription-polymerase chain reaction; GR, glucocorticoid receptor; GAPDH, glyceraldehyde-3-phosphate dehydrogenase; miR-18, microRNA-18; ns, no statistically significant difference.

Next, we evaluated the relationship between GR protein and miR-18 expression levels in the mPFC region of the OVX+Stress mice with or without KSS administration. OVX+Stress mice showed significantly increased miR-18 expression in the mPFC, which was normalized by KSS administration in OVX+Stress+KSS mice (Figure 3a). Moreover, western blot analysis revealed decreased GR protein expression in the mPFC of OVX+Stress mice, whereas it resembled the control level in the OVX+Stress+KSS mice (Figures 3b, c). Chronic stress exposure also decreased the number of GR-immunoreactive cells in the mPFC, which was prevented by KSS administration (Figures 3d, e). Taken together, these findings suggest that KSS reduced miR-18 expression and increased GR protein expression levels by reversing the inhibitory effects of chronic stress exposure on GR protein translation in the mPFC of OVX+Stress mice.

## Discussion

The present study demonstrated that KSS ameliorated GR protein expression changes by downregulating miR-18 expression in the mPFC, which in turn improved unstable emotion-like behaviors and immature morphological changes in the spine formation of pyramidal neurons in the mPFC (Figures 1-3). The mPFC is involved in numerous important cognitive functions, including decision-making, working memory, attention, and emotional control [7]. Several clinical studies have demonstrated the effects of menopause on the mPFC [15]. Menopause is associated with decreased gray matter volume in the mPFC, which may contribute to cognitive changes such as memory problems and difficulties in decision-making [16]. During menopause, there are changes in the levels of hormones such as estrogen, which is involved in the formation and maintenance of synapses and dendritic spines [17]. Furthermore, estrogen replacement therapy increased the density and size of hippocampal dendritic spines in a rat menopause model [18]. These findings suggest that the menopause-related decrease in estrogen levels may be associated with changes in the synaptic function and spine morphology of mPFC neurons. Menopause-related changes in hormone levels may exacerbate the effects of chronic stress. Thus, future studies should focus on understanding the anti-stress mechanisms involved in regulating neuronal functions by the effective chemical components of KSS.

We previously found that Yokukansan, a Japanese herbal medicine, downregulated miR-18 expression and normalized HPA axis activity by regulating GR protein expression in the hypothalamus and corpus callosum of stress-exposed mice. Similarly, our present findings indicated that KSS ameliorated chronic stress-induced unstable emotional behavior and upregulated plasma corticosterone levels (Figure 1). A previous study reported that postmenopausal women had higher levels of cortisol and perceived stress than premenopausal women [19]. However, the molecular mechanisms underlying the changes in cortisol levels and HPA axis activity in postmenopausal women remain unclear. The present study demonstrated that the effects of KSS on the HPA axis involve miR-18 and GR protein expression (Figure 3). Specifically, KSS ameliorated GR protein expression changes by downregulating miR-18 expression in the mPFC, which improved unstable emotion-like behaviors and immature morphological changes in the spine formation of pyramidal neurons in the mPFC (Figure 2).

The limitation of this study is that we did not identify the constituent herbal medicines included in KSS and did not show the function of estrogen receptors (ERs). Among the constituent herbal medicines in KSS, Bupleuri radix is well-known as the main component that may be effective for psychiatric symptoms and could serve as a possible alternative to current antidepressant medicines. Thus, a single administration of Bupleuri radix might show practical antidepressant-like and anti-stress effects in rodents. The next step in our research is to determine the effective chemical components of KSS. Previous studies have reported that miR-18 prevents ERα expression; furthermore, postmenopausal women have shown decreased ERα expression [20]. Therefore, brain miR-18 expression might be crucially involved in GR and/or ERα expression during the onset of menopausal symptoms. However, further studies linking the effective chemical components of KSS to its anti-stress function related to microRNAs in the brain are warranted to clarify the functional implications of these novel findings in the mPFC of chronic stress-exposed postmenopausal model mice.

From a clinical point of view, one previous KSS clinical trial study for postmenopausal women indicated that it was not able to show significant improvement effects in the main survey values [4]. One of the reasons why no significant improvements were found in the main survey values is that this clinical study might include several problems with the study design of participant selection. However, this clinical study also showed that KSS administration for post-menopause women showed significant improvements in excitability and irritability scores [4]. Furthermore, in this study, we indicated that KSS administration showed improvement effects of irritability behaviors for stress-exposed-OVX mice (Figure 1). Furthermore, we found that the manufacturer did not perform the clinical survey of adverse reactions, and the KSS clinical trial study for postmenopausal women for 12 weeks indicated that no serious adverse events were reported [4]. From the results of these clinical trials and our basic research, it is assumed that KSS has a possibility of an effect on improving neuropsychiatric symptoms during menopause, especially irritability.

## Conclusions

In conclusion, KSS administration in chronic stress-exposed postmenopausal mice exerted anti-stress effects and facilitated recovery from immature spine morphologies of basal dendritic pyramidal neurons, at least partly by regulating miR-18 and GR expression in the mPFC.

## Figures and Tables

**Figure 4:**
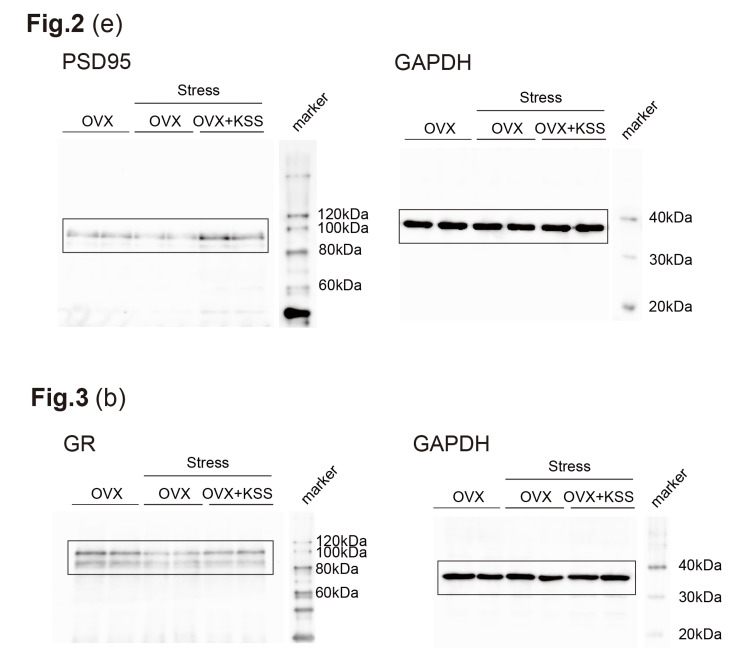
Full-size gels for immunoblots and molecular weight markers in Figures 2, 3. GR, glucocorticoid receptor; PSD95, postsynaptic density protein 95; GAPDH, glyceraldehyde-3-phosphate dehydrogenase; kDa, kilo-Dalton.

## Appendices

**Table 3:** KSS treatment effects on plasma corticosterone levels (Figure 1b) Asterisk (*) and dagger (†) indicate a significant difference between test groups using one-way ANOVA followed by Tukey’s post-test (P = 0.0017 between OVX and OVX+stress, P = 0.0174 between OVX+stress and OVX+stress+KSS groups).

	OVX (n=8)	OVX+stress (n=10)	OVX+stress+KSS (n=10)
Mean	114.8	349.5^*^	182.2^†^
SD	79.1	166.6	107.6

**Table 4:** Number of marbles buried in the marbles burying test (Figure 1c) Asterisk (*) and dagger (†) indicate a significant difference between test groups using one-way ANOVA followed by Tukey’s post-test (P = 0.0009 between OVX and OVX+stress, P = 0.0177 between OVX+stress and OVX+stress+KSS groups).

	OVX (n=17)	OVX+stress (n=18)	OVX+stress+KSS (n=19)
Mean	8.18	12.89^*^	9.53^†^
SD	3.81	2.63	4.17

**Table 5:** Morphological analysis of spines of pyramidal neurons in mPFC using Golgi staining (Figure 2b-d) Spine area (a), length (b), and width (c) of basal dendrites from the cell soma of pyramidal neurons in the mPFC. Asterisk (*) and dagger (†) indicate a significant difference between test groups using one-way ANOVA followed by Tukey’s post-test (*P <0.001, **P <0.0001 between OVX and OVX+stress, ^†^P <0.0001 between OVX+stress and OVX+stress+KSS groups). mPFC: medial prefrontal cortex, OVX: ovariectomized, KSS: Kamishoyosan.

(a)	OVX (n=135)	OVX+stress (n=146)	OVX+stress+KSS (n=260)
Mean	3.59	2.15^**^	3.47^†^
SD	1.28	1.01	1.78

(b)	OVX (n=192)	OVX+stress (n=131)	OVX+stress+KSS (n=261)
Mean	1.88	2.24^*^	1.76^†^
SD	0.92	0.93	0.67

(c)	OVX (n=127)	OVX+stress (n=79)	OVX+stress+KSS (n=157)
Mean	0.90	0.66^**^	1.19^†^
SD	0.32	0.26	0.39

**Table 6:** Densitometric quantification of PSD95 expression in mPFC (Figure 2f) Asterisk (*) and dagger (†) indicate a significant difference between test groups using one-way ANOVA followed by Tukey’s post-test (P = 0.0079 between OVX and OVX+stress, P = 0.0107 between OVX+stress and OVX+stress+KSS groups). mPFC: medial prefrontal cortex, OVX: ovariectomized, KSS: Kamishoyosan.

	OVX (n=7)	OVX+stress (n=7)	OVX+stress+KSS (n=7)
Mean	1.00	0.66^*^	0.99^†^
SD	0.12	0.10	0.28

**Table 7:** Expression levels of miR-18 in mPFC (Figure 3a) Asterisk (*) and dagger (†) indicate a significant difference between test groups using one-way ANOVA followed by Tukey’s post-test (P = 0.0362 between OVX and OVX+stress, P = 0.0146 between OVX+stress and OVX+stress+KSS groups). mPFC: medial prefrontal cortex, OVX: ovariectomized, KSS: Kamishoyosan.

	OVX (n=4)	OVX+stress (n=4)	OVX+stress+KSS (n=5)
Mean	1.00	1.46^*^	0.94^†^
SD	0.07	0.28	0.25

**Table 8:** Densitometric quantification of GR expression (Figure 3c) Asterisk (*) and dagger (†) indicate a significant difference between test groups using one-way ANOVA followed by Tukey’s post-test (P = 0.0123 between OVX and OVX+stress, P = 0.0212 between OVX+stress and OVX+stress+KSS groups). GR: glucocorticoid receptor, OVX: ovariectomized, KSS: Kamishoyosan.

	OVX (n=6)	OVX+stress (n=6)	OVX+stress+KSS (n=6)
Mean	1.00	0.75^*^	0.97^†^
SD	0.27	0.25	0.28

**Table 9:** The relative fluorescence intensity of GR signals in the mPFC (Figure 3e) Asterisk (*) and dagger (†) indicate a significant difference between test groups using one-way ANOVA followed by Tukey’s post-test (P = 0.0022 between OVX and OVX+stress, P = 0.0492 between OVX+stress and OVX+stress+KSS groups). mPFC: medial prefrontal cortex, GR: glucocorticoid receptor, OVX: ovariectomized, KSS: Kamishoyosan.

	OVX (n=3)	OVX+stress (n=4)	OVX+stress+KSS (n=4)
Mean	1.00	0.76^*^	0.88^†^
SD	0.03	0.09	0.04
